# Additive Manufacturing of Vapor Chambers

**DOI:** 10.3390/ma18050979

**Published:** 2025-02-23

**Authors:** Kuan-Lin Chen, Shao-Chi Hsu, Shung-Wen Kang

**Affiliations:** Department of Mechanical and Electro-Mechanical Engineering, Tamkang University, New Taipei City 25137, Taiwan; 811370039@o365.tku.edu.tw (K.-L.C.); 612370030@o365.tku.edu.tw (S.-C.H.)

**Keywords:** vapor chambers, additive manufacturing, triply periodic minimal surface

## Abstract

The increasing power density of high-performance electronic devices poses significant thermal management challenges. Vapor chambers (VCs) offer efficient heat dissipation, but traditional manufacturing methods limit their structural precision and performance. This study investigates the thermal performance of VCs fabricated with additive manufacturing (AM), featuring triply periodic minimal surface (TPMS) Gyroid capillary structures at two fill ratios under varying thermal loads. Enhanced thermal stability and performance were observed in the higher fill ratio, particularly under higher heat loads, whereas the lower fill ratio excelled under low-heat conditions, achieving a thermal resistance as low as 0.3688 K/W at an 80 W heat load. Additionally, the research explored the advantages and challenges of horizontal and vertical printing techniques in VC fabrication. Horizontal printing was found to compromise cavity volume due to necessary support structures, whereas vertical printing enhanced mass production feasibility and maintained effective vapor circulation. This study proposes a novel approach using AM to manufacture VCs as a monolithic structure. By eliminating the need for welding, this method ensures seamless integration of the capillary structure with the housing, thereby avoiding issues related to poor contact or welding-induced damage. The study confirmed a 75% reduction in thermal resistance in VCs with capillary structures compared to those without under similar conditions, highlighting the significant potential of integrating precisely designed capillary structures and additive manufacturing in improving vapor chamber performance for advanced thermal management applications.

## 1. Introduction

With the rapid advancement of artificial intelligence (AI) technologies, the computational performance of high-power components such as central processing units (CPUs) and graphics processing units (GPUs) continues to increase. Modern AI accelerators, such as the NVIDIA GB200, have significantly pushed power consumption boundaries, with some high-performance GPUs reaching over 1000 watts per package in data center configurations. Similarly, server-grade CPUs and AI processors have thermal design power (TDP) at the package level exceeding 500 watts per package in conventional water-cooled systems [1], with HPC packages surpassing 900 watts per package under advanced two-phase immersion cooling solutions [2]. This escalating power demand imposes significant challenges on thermal management systems, requiring more advanced cooling solutions to ensure the operational stability of devices and support additional applications. Among various cooling technologies, vapor chambers (VC) have emerged as a highly efficient and reliable thermal management component, capable of quickly transferring heat from a localized area and evenly dispersing it over a larger surface.

**Figure 1:**
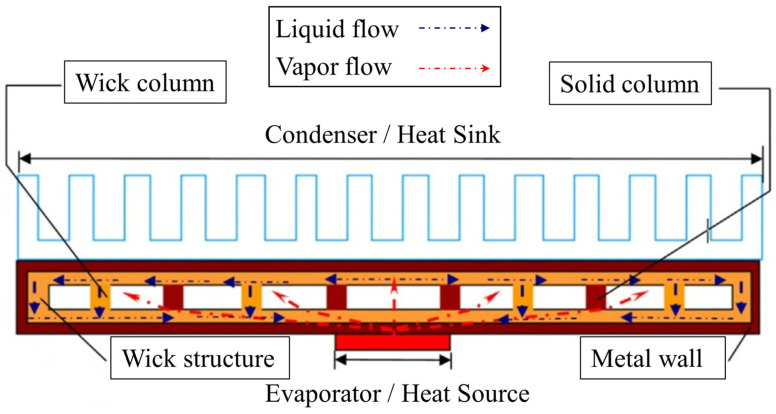
Working principle and structure of vapor chambers.

The core functionality of vapor chambers lies in their ability to rapidly, stably, and uniformly transfer heat from the heat source. At the heart of this process is the capillary structure, which plays a crucial role in facilitating effective thermal management. The capillary structure consists of microscopic channels that utilize capillary forces to drive the working fluid back to the evaporator end, forming a complete thermal transfer cycle, with the detailed cycle and structure shown in Figure 1. In this cycle, the working fluid absorbs heat at the evaporator and evaporates into vapor, which quickly flows to the condenser. At the condenser, the vapor releases heat and condenses back into a liquid. The liquid is then returned to the evaporator through the capillary structure, completing the cycle without requiring external power.

The design and quality of the capillary structure have a direct and critical impact on the thermal performance of VC. The microstructure, including pore size, distribution density, and surface roughness, not only affects the return flow rate of the working fluid but also determines the thermal resistance and overall cooling efficiency of the VC. Previous studies have demonstrated that optimizing the design of the capillary structure can significantly enhance the performance of vapor chambers, especially in thermal management applications for high-power heat sources [3].

Capillary structures play a crucial role in VC, as their microscopic channels generate capillary forces that facilitate the circulation of the working fluid between the evaporator and condenser. This significantly enhances heat transfer efficiency. Consequently, improving capillary structure design has become a primary focus for enhancing VC performance.

Previous studies have introduced various innovative capillary structure designs. For instance, Tang et al. [4] developed a composite wick structure combining micro V-shaped grooves with sintered copper powder. They found that this structure exhibited significantly greater capillary force compared to using grooves or sintered structures alone. This improvement was attributed to the wetting interaction between the grooves and sintered powder, which provided an additional source of capillary force. Similarly, Deng et al. [5] proposed a composite porous vapor chamber (CPVC) featuring uniform radial grooves at the evaporator and sintered copper powder of varying sizes in different sections. Experimental results demonstrated that the CPVC effectively reduced surface temperature differences and achieved a 10% to 130% reduction in thermal resistance compared to pure copper plates.

In another study, Liu et al. [6] fabricated a vapor chamber with sintered multi-artery structures, where the evaporator featured columnar and arterial designs. Different particle sizes of sintered copper powder were used at specific locations. This design achieved an exceptionally low thermal resistance of 0.016 K/W, highlighting the benefits of increased porosity and surface area in enhancing capillary structure performance. Meanwhile, Zeng et al. [7] developed a grooved structure VC with a micro-groove cavity array (MGRA) consisting of high-aspect-ratio microchannels and cavities. This design enhanced capillary forces and facilitated fluid phase change. It achieved a thermal resistance of 0.074 K/W under horizontal testing and maintained a low thermal resistance of 0.055 K/W at a 30° tilt, showcasing excellent anti-gravity capabilities and stability.

Optimizing the geometry of capillary structures has also been a significant research direction. Li et al. [8] applied the Entransy Dissipation Rate (EDR) theory to design vein-like groove structures. These structures substantially increased capillary pressure and improved temperature gradients, further enhancing the thermal performance of heat spreaders. Similarly, Zhou et al. [9] developed a multi-artery VC with sintered copper powder structures and solid copper columns, achieving fast thermal response, high heat flux limits, and a low thermal resistance of 0.033 K/W.

While these studies have achieved significant progress in improving capillary structure design and performance, they remain constrained by traditional manufacturing techniques. Conventional methods such as mechanical machining, sintering, and joining techniques (e.g., soldering [10], high-frequency welding [5], and diffusion bonding [3]) often suffer from limitations in precision and design flexibility. Uneven distribution of internal capillary structures can reduce heat transfer efficiency, while additional machining or assembly steps may cause structural damage or poor contact between the chamber and housing, further impacting reliability and performance.

Future research should focus on emerging manufacturing technologies such as AM. AM offers higher precision and design flexibility, enabling the fabrication of complex capillary structures while reducing manufacturing difficulty and cost. This approach has the potential to overcome the limitations of traditional techniques, paving the way for the development of highly efficient VC. These innovations are expected to play a critical role in addressing the growing demand for high-power-density and precise thermal management solutions.

AM is an innovative production technology that fabricates objects directly from 3D models, fundamentally differing from traditional manufacturing methods. In AM processes, after simplifications through slicing and support design, 3D CAD files can generate complex components that closely resemble the final product. This approach eliminates the need for the time-consuming steps of geometric feature analysis, tool selection, and machining techniques required in conventional manufacturing.

One of the key advantages of AM over traditional methods is its ability to create highly intricate geometries and internal structures without relying on tool paths [11] or the assembly of multiple parts. This capability has significant potential in thermal management, where it can overcome performance bottlenecks in existing heat transfer systems. However, AM technology still faces several challenges, including poor surface roughness, large tolerances, powder removal and cleaning difficulties, support structure requirements, and issues related to the strength of finished products and the complexity of post-processing [12].

Recent advancements in metal AM have facilitated the reliable fabrication of complex geometries and gradient designs, improving contact performance and enabling integrated designs that closely match simulation results. AM-fabricated capillary structures exhibit superior order compared to the random formations produced by sintering or metal foaming, significantly enhancing heat transfer device performance [13].

AM’s capability to produce intricate components minimizes material waste and supports various metals, including titanium, aluminum, and stainless steel [14]. Copper, while challenging due to high reflectivity and thermal conductivity, has become more feasible with advancements in powder oxidation resistance, alloyed powders, and increased laser power. Aluminum and titanium alloys offer lightweight and corrosion-resistant benefits but require argon gas during processing, increasing costs. However, as conventional methods struggle with sintered capillary structures, AM plays an irreplaceable role in developing porous structures and related technologies [15,16].

Triply periodic minimal surface (TPMS) structures, derived from mathematical and physical theories, minimize surface tension to achieve local equilibrium. These structures exhibit high symmetry, zero average curvature, and periodicity in three dimensions. Their presence in nature, such as butterfly wing microstructures [17] and weevil exoskeletons [18], highlights their stability and energy efficiency, contributing to superior mechanical performance.

TPMS structures align seamlessly with 3D printing, allowing precise replication of complex geometries. This synergy enables high-strength, low-weight materials, offering innovative solutions for engineering challenges. By leveraging TPMS and AM, engineers can develop advanced components that meet stringent performance standards while reducing material consumption and production complexity, broadening TPMS applications across various fields [19].

The use of AM for fabricating VC remains a relatively nascent area of research, with only a handful of studies exploring this innovative manufacturing technique. Ozguc et al. [20] employed direct metal laser sintering (DMLS) to fabricate a VC using 316L stainless steel, comparing its thermal performance with that of a solid metal block of the same material. The results demonstrated the superior thermal performance of the VC, establishing the feasibility of using 3D printing for VC production. Similarly, Meng et al. [21] developed an AMVC featuring a composite porous structure. The AMVC incorporated a framed evaporator, TPMS Schwarz P-structured support columns, and a TPMS Gyroid-structured condenser, validating its performance through extensive experimentation.

Gu et al. [22] extended this approach by fabricating aluminum VCs with grooved porous structures using AM. Comparative experiments revealed that grooved porous VCs exhibited lower thermal resistance and higher temperature uniformity than their groove-only counterparts. Building on this, Gu et al. [23] fabricated a VC with a TPMS Gyroid porous structure, showing that this design significantly enhanced liquid return by creating efficient microchannels for liquid flow, thereby improving thermal performance.

Despite these advancements, existing studies commonly rely on a segmented manufacturing approach, where the VC structure and housing are fabricated separately and then joined through welding. This approach introduces several challenges, including poor contact between the capillary structure and the housing, as well as structural damage during the welding process. These issues limit the overall reliability and performance of the final product.

To address these limitations, this study proposes a novel approach using AM to manufacture VCs as a monolithic structure. By eliminating the need for welding, this method ensures seamless integration of the capillary structure with the housing, thereby avoiding issues related to poor contact or welding-induced damage. The proposed VC design incorporates a TPMS Gyroid capillary structure with a porosity of 60%, and two fill ratios (100% and 150%) are investigated. Additionally, a control VC without any capillary structure is fabricated for comparison.

The proposed all-in-one VC design not only addresses the shortcomings of traditional segmented manufacturing but also leverages the flexibility of AM to integrate advanced porous structures. The TPMS Gyroid capillary structure further enhances liquid return efficiency, improving thermal performance while maintaining structural robustness. This study demonstrates the potential of AM to revolutionize VC fabrication, offering a more reliable and efficient solution for next-generation thermal management systems.

## 2. Vapor Chamber Design and Additive Manufacturing Experiment

### 2.1. Vapor Chamber Design

We used the Tongtai AMP-160 to print our samples and selected stainless steel (316L) as the material for printing. Table 1 outlines the operational parameters of the AMP-160 machine. The manufacturing mode of this machine is selective laser melting (SLM), which is a type of additive manufacturing (AM) technology. SLM utilizes a high-power-density laser to melt and fuse metallic powder. Components are built by selectively melting and fusing the powder within and between layers [24]. When selecting the printing parameters, we referred to previous research [25], which indicates that laser power significantly affects the metal’s density and dimensional accuracy. Therefore, we used different parameters for printing the walls and the internal porous structure of the VC, as shown in Table 2 below.

This study examines the VC design by dividing it into three key components: the condenser, support columns, and evaporator. The design approach emphasizes integration and optimization of these components to improve thermal performance and operational efficiency.

The condenser and support columns were designed as an integrated structure rather than separate entities. The condenser’s functionality was incorporated into the top space of the VC, with the top surface acting as the condensation area. To enhance condensation efficiency, a TPMS Gyroid structure was employed within the support columns, introducing porosity to facilitate effective thermal management. This configuration allows vapor to condense efficiently at the top surface, while the porous structure aids in wicking the condensate back to the evaporator, ensuring continuous fluid circulation.

For the porous structure, a mixed approach was adopted by combining TPMS Gyroid geometries with specific design adjustments for the evaporator and support column regions. The porosity of the structure was fine-tuned by modifying parameters such as unit cell size and wall thickness. These adjustments enable precise control over the porosity, ensuring the structure provides optimal liquid return capabilities while maintaining structural integrity.

This integrated design approach, leveraging TPMS structures, highlights the synergy between advanced geometric modeling and AM. By unifying the condenser and support columns into a cohesive design, this study demonstrates an innovative strategy for enhancing the thermal performance and reliability of VC in demanding thermal management applications.

For the evaporator, much of the VC performance is determined by the capillary structure. Its primary function is to use capillary force to quickly return the condensed liquid from the condenser to the evaporator, thereby completing a stable thermal cycle. This capillary return process is crucial to the performance of the VC. Without the capillary structure, the liquid cannot flow back, leading to a lack of liquid replenishment within the VC, which would negatively impact the evaporation efficiency and thermal conductivity.

Secondly, the capillary structure plays a key role in the temperature uniformity within the VC. The operation of the VC requires the formation of a uniform temperature field inside to prevent localized overheating. By increasing the liquid return speed, the capillary structure ensures heat exchange balance between the evaporative and condensing regions, reducing the temperature gradient. This is particularly important for applications in high-heat-flux environments, where any temperature non-uniformity could result in equipment failure or decreased performance.

Moreover, the design of the capillary structure directly affects the thermal resistance of the VC. Thermal resistance is a critical indicator of the VC’s performance, and lower thermal resistance translates into higher heat dissipation efficiency. By optimizing the shape of the capillary structure, the thermal resistance within the VC can be significantly reduced, thereby improving overall heat dissipation capability. Factors such as the choice of capillary structure material, pore size, and porosity all influence thermal resistance. For example, finer capillary pores can provide stronger capillary forces, facilitating faster liquid return. However, overly dense structures may increase fluid resistance, which could hinder liquid flow. Therefore, these factors must be balanced during the design process.

**Figure 2:**
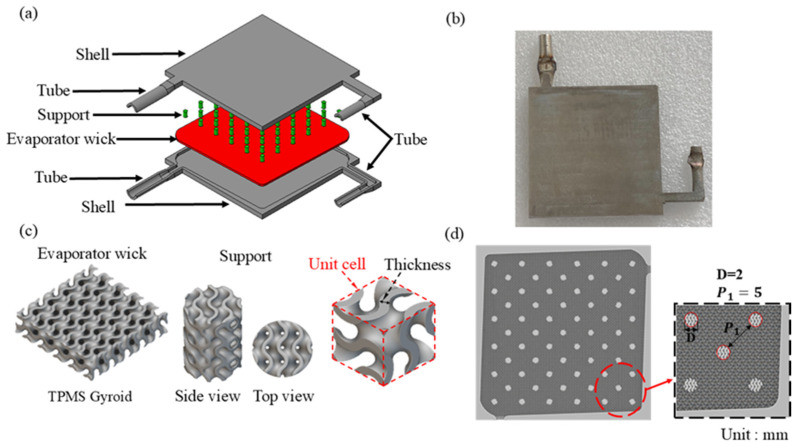
Additive manufacturing of vapor chambers: (**a**) exploded view, (**b**) solid model, (**c**) evaporator wick and support columns, and (**d**) support columns distribution.

In this study, the VC has dimensions of 62.5 × 62.5 × 5 mm^3^ with two pipes located on the bottom and right side for powder removal and working fluid filling, respectively. Using TPMS, Gyroid structures were designed for the evaporator, with porosity settings of 60%. Based on the conclusions drawn from the support design and printing strategy, we ultimately designed the support columns as 2 mm diameter cylindrical structures. These were integrated with the TPMS Gyroid porous structure to balance the working fluid’s return capability with the structural strength, enabling the support columns to wick liquid back to the evaporator. The supports were uniformly distributed within the VC with a spacing of 5 mm. The overall structural design and parameters are shown in Figure 2 and Table 3.

### 2.2. Manufacturing Directions of Vapor Chambers

In AM processes, the layer-by-layer stacking method results in a direct correlation between the height of the component and the printing time. To improve production efficiency and reduce printing time, the initial design opted for horizontal printing of the VC, aiming to lower height requirements and achieve faster manufacturing. However, horizontal printing introduces additional challenges due to gravitational effects, increasing the need for support structures and causing a series of manufacturing issues.

**Figure 3:**
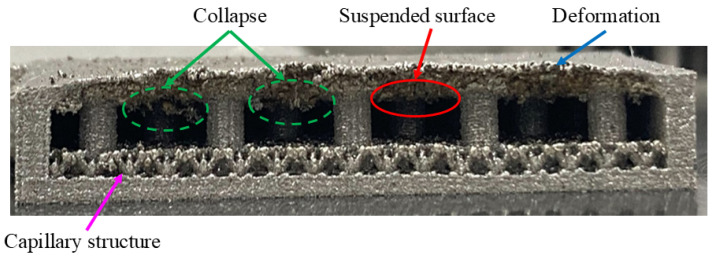
Defect issues caused by horizontal printing.

During horizontal printing, excessive unsupported surfaces (suspended surface) may form within the component, especially when the support structures are insufficient. These unsupported surfaces are prone to collapse during printing, leading to print failures. Even if printing is completed, the component surface may exhibit significant defects caused by gravitational effects and uneven stress distribution, such as edge warping, structural deformation, collapse, or localized damage (as shown in Figure 3). These defects not only affect the appearance of the VC but also severely compromise its functionality and reliability, further reducing its thermal performance and durability.

To address the issues associated with horizontal printing, the design incorporated increased numbers and sizes of support structures to stabilize the component. However, enlarging the diameter of the supports inevitably reduces the internal cavity volume of the VC, diminishing the effective space for working fluid circulation. While this modification improves printing stability, it negatively impacts the thermal performance of the VC, lowering its operational efficiency.

In summary, although horizontal printing can reduce manufacturing time, it presents significant limitations in practical applications. If machine cost factors necessitate horizontal printing, future efforts should focus on optimizing printing parameters and support structure design to balance structural stability with internal functional space. Exploring more efficient and reliable printing strategies is crucial to meeting functional requirements and ensuring manufacturing feasibility.

**Figure 4:**
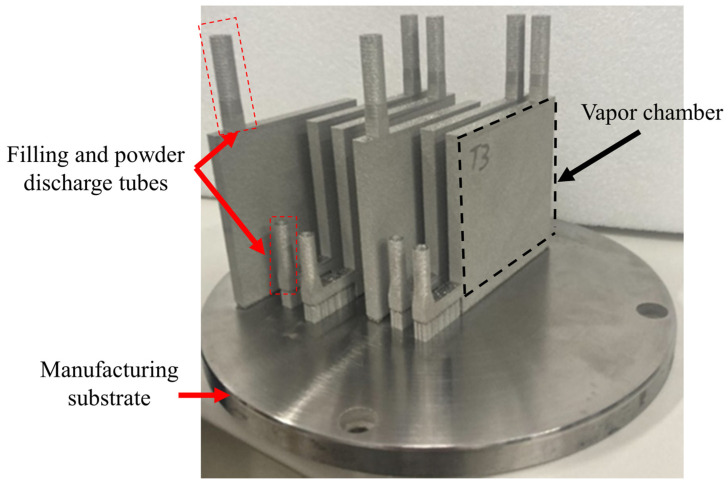
Using vertical printing for vapor chambers.

In the design of VCs, sufficient vapor space is a critical factor for ensuring efficient operation. To maximize the internal cavity volume and enhance performance, this study ultimately adopted vertical printing, as illustrated in Figure 4. Vertical printing offers several advantages, making it the optimal choice to meet the design requirements of VCs.

The primary advantage of vertical printing lies in its ability to avoid the creation of large overhanging structures. During the printing process, all areas receive some degree of support, which effectively reduces the risk of structural collapse and helps maintain the overall integrity of the VC. Additionally, vertical printing reduces the number of required support structures, thereby increasing the cavity volume between the evaporator and condenser ends. This improvement enhances the circulation efficiency of the working fluid and contributes to the overall thermal performance of the VC.

Furthermore, vertical printing shortens post-processing time. After printing, the VC needs to be cut from the substrate, and since vertically printed VCs have a smaller contact area with the substrate, the cutting process requires less time, thereby improving production efficiency.

In summary, vertical printing demonstrates significant advantages in maintaining structural stability, maximizing vapor space, and optimizing production efficiency. This method not only meets the design requirements of VCs but also provides valuable insights for the future application of AM technologies in thermal management components.

**Figure 5:**
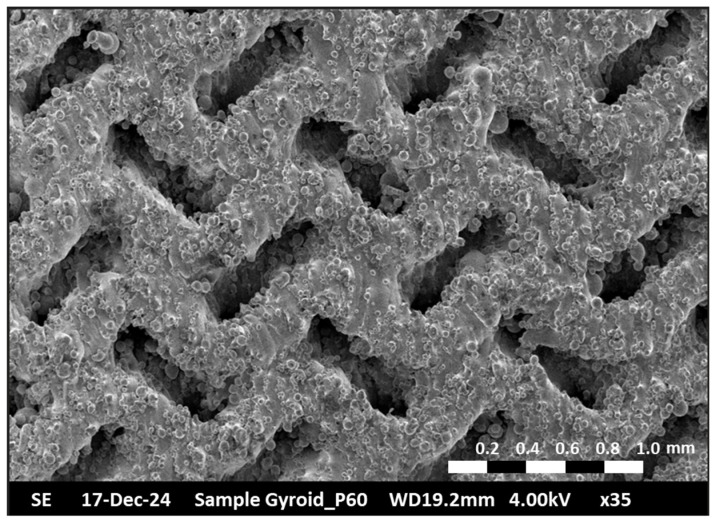
SEM imaging of a sample of TPMS Gyroid wick.

In this study, the TPMS Gyroid structure was selected as the capillary structure for the VC, with a porosity of 60%. To evaluate its structural integrity and feasibility, a capillary sample (10 mm × 10 mm × 10 mm) was fabricated and analyzed using scanning electron microscopy (SEM). The SEM analysis was conducted using a HITACHI S-2600N (Tokyo, Japan) at an accelerating voltage of 4–10 kV and a working distance of 10–20 mm, with images captured at a magnification level of approximately 30 to 40 times under high-vacuum conditions. Prior to imaging, the samples underwent ultrasonic cleaning followed by air drying to remove surface contaminants, and the analysis was performed at a controlled temperature of 18 °C. As shown in Figure 5, the SEM results reveal that the structure’s surface exhibits fine granular features, which effectively enhance capillary forces and facilitate the return of the working fluid. Furthermore, the overall structure remained intact, with no signs of damage or incompleteness, confirming that the TPMS Gyroid structure offers excellent stability and reliability for VC design and fabrication.

### 2.3. Post-Processing and Filling of Vapor Chambers

The VCs are fabricated using the SLM process, where layers of metal powder are melted and stacked sequentially to create the desired structure. This process involves using a high-energy laser to melt metal powder particles layer by layer. However, the nature of the SLM process often results in rough and uneven surfaces on the finished product. Several factors contribute to this surface roughness. During the melting process, incomplete melting of some powder particles can occur due to uneven laser energy distribution, leaving residual particles adhered to the surface. Additionally, the solidification characteristics of the laser melt pool play a significant role. As the molten metal cools and solidifies, surface tension and thermal stresses can cause irregular surface patterns, further increasing roughness. The layered deposition also contributes to surface irregularities, as excess powder may be redeposited onto the surface by the molten metal during the stacking process. Furthermore, the scanning path design and heat accumulation in certain areas can lead to micro-defects, such as pores and uneven melting, which exacerbate the surface roughness.

In our previous studies, we have confirmed the influence of laser parameters on the fabrication of 316L stainless steel [25], ensuring that the printed chambers possess sufficient density and maintain airtightness. These inherent characteristics of the SLM process result in surfaces that are not only rough but also inconsistent, which significantly impacts performance in practical applications. A rough surface increases contact thermal resistance, thereby reducing heat transfer efficiency. To address these issues, post-processing is essential. Grinding is employed to smooth the surface of the printed VCs, improving both flatness and uniformity.

**Figure 6:**
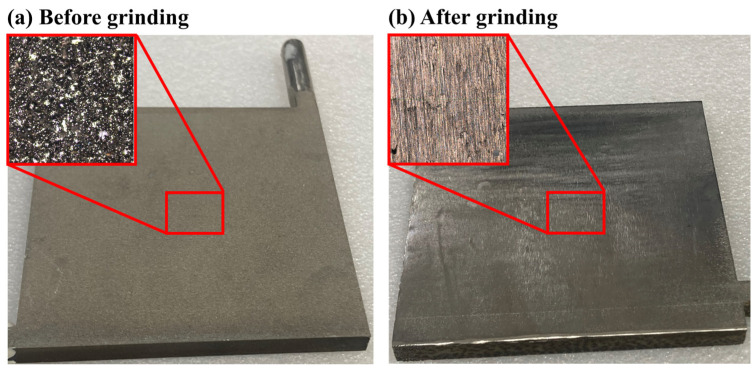
Actual surface of vapor chamber captured with confocal microscope (**a**) before grinding and (**b**) after grinding.

Figure 6a,b demonstrate the differences in surface morphology before and after surface treatment. Using a confocal microscope, the surface was analyzed, and measurements were collected. The results showed that key surface roughness parameters, including $S_{a,avg}$,$S_{q,avg}$, $S_{p,avg}$, $S_{v,avg}$, and $S_{z,avg}$, significantly decreased after grinding, as shown in Table 4. Furthermore, the variation in these parameters across the processed VCs was controlled to within 10%, ensuring consistency among samples. The reduction in these parameters indicates that the surface became smoother, more uniform, and flatter after grinding, effectively reducing thermal resistance and enhancing heat transfer performance. These findings highlight the critical role of surface post-processing in improving the thermal efficiency of 3D-printed VCs.

**Figure 7:**
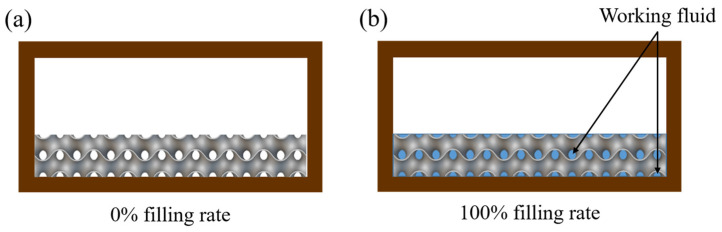
Filling ratio schematic for the wick: (**a**) 0% filling ratio, (**b**) 100% filling ratio.

The working fluid used in the experiment was methanol. During the preparation phase, a vacuum pump was employed to evacuate the chamber to below $1\times 10^{-2}$ Torr, ensuring that any residual air was removed. Once the desired vacuum level was achieved, a precise quantity of methanol was injected into the system to establish the targeted fill ratio. A 100% fill ratio is defined as the condition where the capillary structure within the evaporator is completely saturated with the working fluid, as illustrated in Figure 7.

To accurately calculate the pore volume of the porous structure and the corresponding working fluid filling amount, this study utilized nTop software (Version 5.16.2) for analysis. The fill ratios and their corresponding fluid filling amounts and subsequent abbreviations are summarized in Table 5. For example, a porosity of 60% with a fill ratio of 100% is referred to as P60F100, while a porosity of 60% with a fill ratio of 150% is referred to as P60F150. The filling amount for the without-wick VC is based on P60F100 and is referred to as WVC. This step ensures consistency and reproducibility across all experimental conditions.

To evaluate the performance enhancement provided by the capillary structure, an additional VC of identical dimensions but without a capillary structure was fabricated. This control VC was filled with the same quantity of methanol as a 100%-filled VC with a capillary structure. This comparison allowed for isolating the effect of the capillary structure on thermal performance.

In the experimental setup, the VC with and without the capillary structure were subjected to identical testing conditions. The impact of the capillary structure on heat transfer performance, including thermal resistance and temperature uniformity, was analyzed based on the recorded data. This methodological approach ensures that the observed differences in performance can be directly attributed to the presence of the capillary structure, providing a robust basis for evaluating its effectiveness in thermal management applications.

## 3. Heat Transfer Experiments of Vapor Chambers

**Figure 8:**
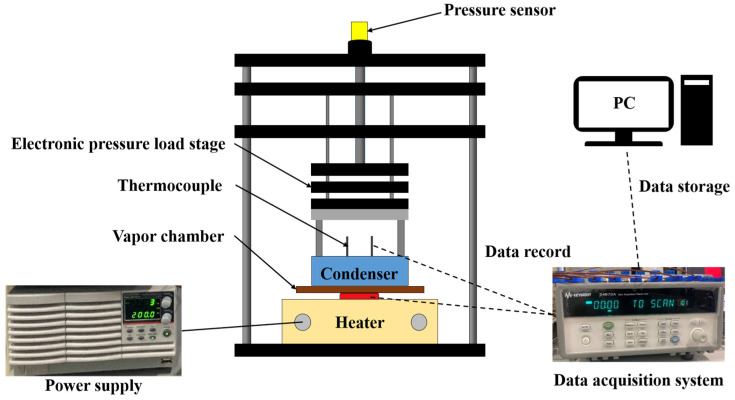
Schematic diagram of the experimental platform.

**Figure 9:**
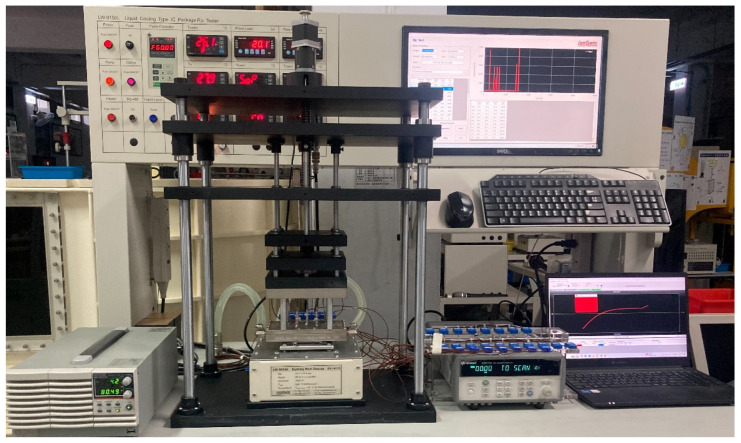
Actual image of the experimental platform.

**Figure 10:**
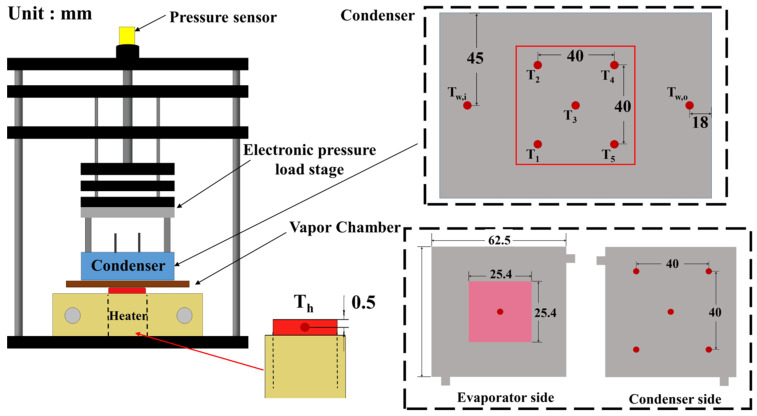
Temperature measurement points of the experiment.

The experimental setup is shown in Figure 8 and Figure 9, while Figure 10 clearly indicates the temperature measurement points and dimensions. The power of the heater is controlled using a GW Instek PSW 80–40.5 power supply (New Taipei City, Taiwan), with a maximum output of 1000 W. During the design of the heater, it was ensured that the power output could be fully transferred to the VC, with power loss controlled within 1%. The condenser is maintained at a stable temperature using a circulating water cooling system. During the testing process, the temperature at the inlet and outlet of the cooling water is measured, ensuring the temperature difference remains within 1 °C to ensure the condenser’s temperature can be considered uniform. If the temperature difference exceeds 1 °C, the cooling water flow rate is adjusted for control. The VC will be subjected to a fixed pressure of 20 kgf using an electronic pressure loading platform to ensure stable contact with both the condenser and the heater. Additionally, an L-shaped spacer is designed for positioning to ensure that each test is conducted at the same location with the same pressure. After completing the test, temperature data are recorded using the KEYSIGHT 34972A data acquisition system (Santa Rosa, CA, USA), which is then transmitted to a computer for further analysis to evaluate the performance.

The temperatures on the condenser side (labeled T_1_~T_5_) are measured using a special design where the measurement points directly contact the condenser side surface, rather than embedding thermocouples into the sides. This ensures that the contact between the VC and the condenser is not affected by wires or grooves. The surface temperature of the heating load (T_H_) is measured 0.5 mm below the heating surface, accurately simulating the temperature conditions of actual thermal loads such as chips. These temperature measurement points provide critical temperature data during the heating and condensing processes, facilitating process analysis and the final calculation of thermal resistance.

An electronic pressure loading platform was utilized to apply a fixed pressure of 20 kgf, ensuring consistent and stable contact between the VC, the heater, and the condenser throughout the testing process. To maintain temperature uniformity at the condenser, the cooling plate’s water flow rate was set at 20 LPM, and the water temperature was precisely controlled at 28 °C. These conditions ensured uniform cooling and consistent experimental settings for all tests, minimizing potential variances.

During the experimental phase, the heater power was systematically increased in increments, starting at 20 W and progressing through 40 W, 60 W, 80 W, 100 W, and 120 W, reaching a maximum of 140 W. When the temperature fluctuation remained within ±0.2 °C over an extended period, it was considered to have reached a stable state, and the next power level was tested, ensuring that steady-state temperature data were captured. Additionally, to ensure operational safety and simulate realistic thermal load conditions, a maximum temperature threshold of 100 °C (T_h,max_) was established. If the heater temperature exceeded this limit, the experiment was immediately terminated, and the data for this power level were not recorded to prevent system damage or data distortion. Experimental settings are organized in Table 6.

The recorded temperature data were subsequently used to calculate the thermal resistance, a key parameter for evaluating the thermal performance of the system. Thermal resistance quantifies the system’s ability to dissipate heat and is a critical metric in thermal management studies. The thermal resistance (R_system_) was determined using Equation (1):

(1) $$R_{system}=\frac{T_{h}-T_{c,avg}}{Q}$$

where *T_h_* represents the surface temperature of the heater, *T_c,avg_* the average temperature of the condenser, and *Q* the heat input. This method provides a quantitative basis for comparing the thermal performance under varying operational conditions.

## 4. Results and Discussion

The condenser in this study is designed to handle a maximum thermal load of 1000 W. However, the experimental maximum thermal load was limited to 140 W due to the restriction of the heater surface temperature to 100 °C. Under these conditions, the condenser’s cooling capacity was sufficient to dissipate the heat supplied by the heater. The following sections analyze the temperature profiles of different samples (WVC, P60F100, and P60F150), focusing on the impact of capillary structures on vapor chamber performance.

### 4.1. Temperature Profile Analysis of WVC

**Figure 11:**
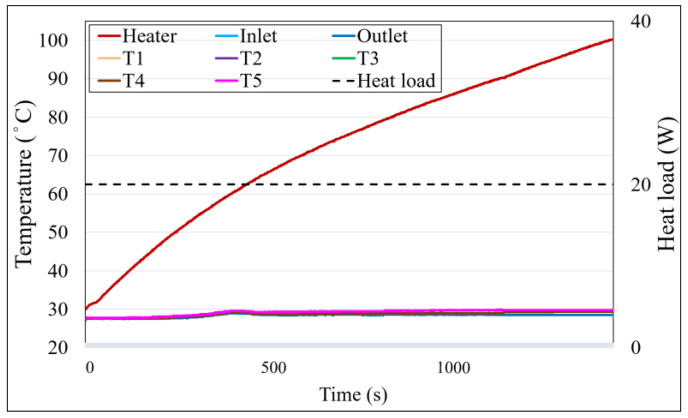
Temperature profile of WVC.

Figure 11 illustrates the temperature profiles of the cooling water inlet and outlet, the condenser side temperatures (T_1_ to T_5_), and the heater temperature (T_H_). The applied heating power is marked with a black dashed line, and the corresponding values are indicated on the secondary axis. The results show that the heater temperature continuously increases after heating begins. Although the temperature gradient slows down over time, the heater temperature reaches and exceeds the experimental equipment’s limit of 100 °C at approximately 1400 s. To ensure equipment safety and maintain experimental consistency, the test was paused at this point, preventing the acquisition of actual steady-state data. Furthermore, the condenser side temperatures are almost identical to the cooling water temperatures, highlighting the limitations of WVC without a capillary structure.

In the absence of a capillary structure, the contact area between the fluid and the wall is significantly reduced, leading to an unclear boiling state and lower overall heat transfer efficiency. Additionally, the high cooling capacity of the condenser ensures that the condenser side temperatures remain nearly equal to the cooling water temperatures. These findings indicate that WVC lacks the necessary thermal performance to support efficient heat cycling.

### 4.2. Temperature Profile Analysis of P60F100

**Figure 12:**
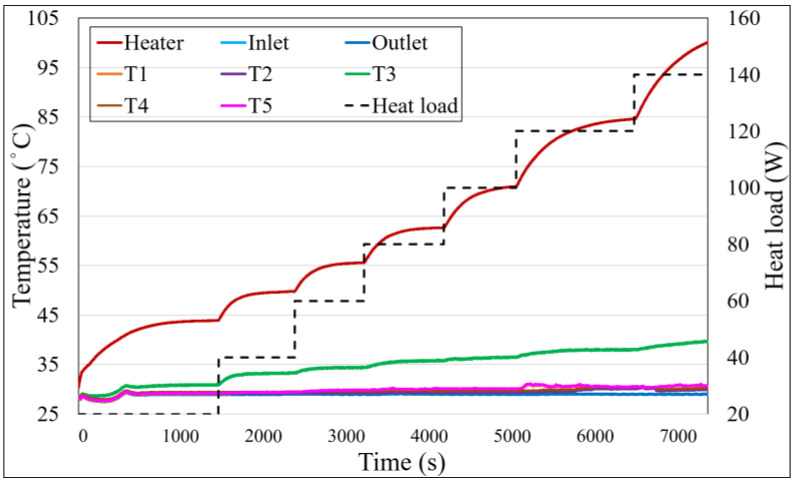
Temperature profile of P60F100.

Figure 12 illustrates the temperature profile of P60F100, which demonstrates significant improvements compared to WVC. At a heating power of 20 W, P60F100 maintains a stable temperature of approximately 44 °C, allowing the experiment to progress to higher power levels. Ultimately, the heater temperature exceeds the limit at 140 W, indicating that the maximum heating power for this sample is 120 W. Observing the condenser side temperatures, it is evident that, compared to WVC, all temperatures increase slightly, with T_3_ showing the most pronounced rise, while other edges only increase by 2–3 °C. At low heating powers, minimal changes are observed, suggesting that evaporation and condensation primarily occur in the central region of the vapor chamber.

This phenomenon is due to the heater’s area being smaller than the VC’s surface area, causing heat transfer to the vertical top to cover shorter distances than radial heat transfer to the sides. Consequently, at low heating powers, most of the heat is managed by the capillary structure in the central region. Additionally, the low thermal conductivity of 316L stainless steel amplifies the distance factor. At higher heating powers, more noticeable temperature changes occur across the condenser side, except for T_3_. However, as the heating power increases, the incremental rise in steady-state temperatures becomes more significant, indicating a decline in performance. This suggests that the thermal flux in the central region becomes insufficient to dissipate all the heat supplied by the heater, causing excess heat to spread outward. While this allows larger areas of the capillary structure to assist in heat dissipation, the low thermal conductivity of the material may limit overall heat transfer performance.

### 4.3. Temperature Profile Analysis of P60F150

**Figure 13:**
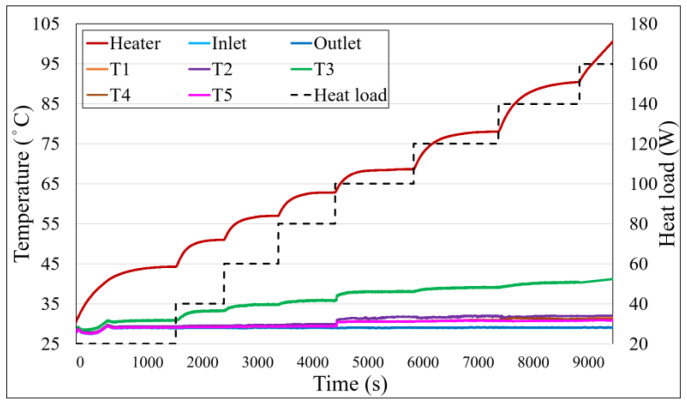
Temperature profile of P60F150.

Figure 13 illustrates the temperature profile of P60F150, which reveals an improved performance compared to P60F100, with the maximum power handled increasing to 140 W. This enhancement is attributed to the higher fill ratio, which provides more working fluid to accommodate greater thermal loads. At low heating powers, only the central T_3_ region shows a significant temperature rise. However, as the heat load increases to 100 W, all condenser side temperatures exhibit noticeable increases, indicating behavior similar to P60F100. Nevertheless, the higher fill ratio in P60F150 mitigates the impact of insufficient fluid return, ensuring that thermal performance does not degrade as significantly. These results demonstrate that increasing the fill ratio effectively enhances the thermal capacity of the vapor chamber and improves overall heat transfer performance.

### 4.4. Temperature Profile Analysis of Three Vapor Chambers Under Different Heat Loads

**Figure 14:**
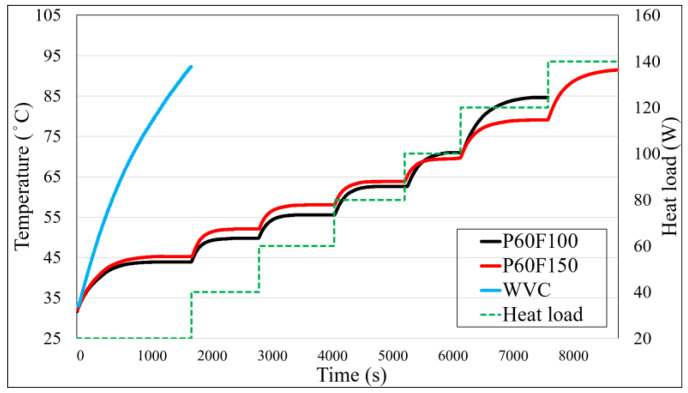
Combined temperature curve for all samples.

Figure 14 illustrates the temperature profiles of three VCs under varying heat loads. For simplicity, only the heater temperature is shown. The data reveal significant differences in heat transfer performance among the samples as the heat load increases.

For WVC, the temperature profile indicates that it fails to reach a steady state even at a low heat load of 20 W, highlighting its poor heat transfer performance and inability to sustain a stable evaporation-condensation cycle. In contrast, P60F100 and P60F150 exhibit much better thermal performance. Within the 20 W to 80 W heat load range, P60F100 consistently achieves lower steady-state temperatures compared to P60F150. This is because, at low heat loads, the evaporation rate remains relatively low, allowing a smaller amount of working fluid to effectively maintain the evaporation-condensation cycle. Additionally, the lower thermal capacity of P60F100 enhances its heat transfer performance, resulting in reduced steady-state temperatures.

However, as the heat load increases to 100 W, the temperature gradient of P60F100 begins to rise more sharply, and its steady-state temperature exceeds that of P60F150. This difference becomes even more pronounced at a heat load of 120 W, illustrating a decline in P60F100’s heat transfer capacity under high heat load conditions. Ultimately, at 140 W, P60F100 exceeds the predetermined temperature limit, indicating its performance limitations at higher heat loads.

In contrast, P60F150 demonstrates greater stability under higher heat loads. Although its temperature gradient also increases at 120 W, its higher fill ratio effectively enhances its maximum heat load capacity, enabling better heat dissipation performance.

Overall, the low-fill sample (P60F100) exhibits superior thermal performance at low heat loads but gradually loses this advantage as the heat load increases. Conversely, the high-fill sample (P60F150) displays improved stability and performance under high heat loads. These findings indicate that fill ratio plays a critical role in determining the thermal performance of VC and should be carefully optimized based on specific application requirements.

### 4.5. Thermal Resistance Analysis and Performance Evaluation of Different Vapor Chambers

**Figure 15:**
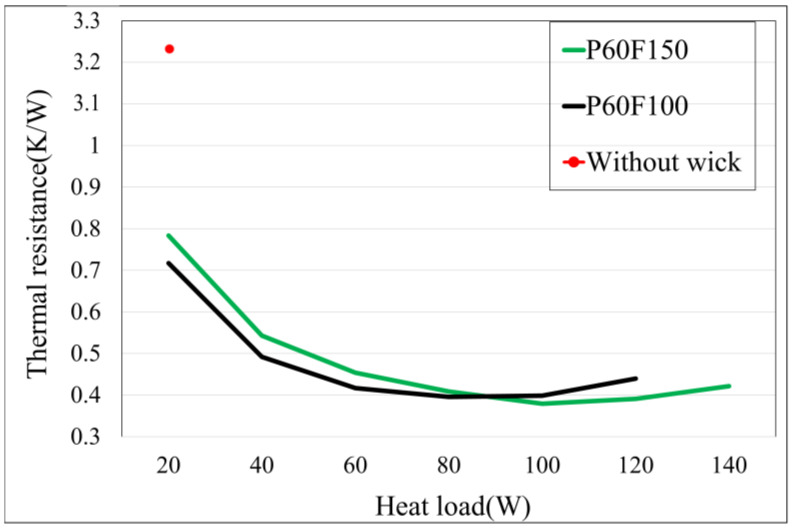
Combined thermal resistance plot for all samples.

Figure 15 illustrates the comprehensive thermal resistance graph, revealing that WVC exhibits poor performance, with a thermal resistance as high as 3.23 K/W, indicating its significant limitations in thermal management. In contrast, P60F100 demonstrates lower thermal resistance under low heat loads, reaching a minimum value of 0.3688 K/W at a heat load of 80 W. As the heat load increases, the thermal resistance gradually rises, consistent with the previously observed increase in condenser side temperatures, which indicates a decline in thermal performance. This trend continues until P60F100 reaches its maximum heat load at 120 W.

P60F150 maintains good thermal performance even under high heat loads. As observed in the temperature profiles, when the heat load increases to 100 W, the condenser side temperatures exhibit noticeable changes, and the thermal resistance reaches its minimum value of 0.3793 K/W. Due to the higher fill ratio, P60F150 is able to sustain a similar level of thermal resistance at a heat load of 120 W. However, when the heat load increases to 140 W, the thermal resistance rises significantly but remains relatively stable and effective in managing heat dissipation. While P60F150 exhibits slightly higher thermal resistance than P60F100 under low heat loads, it shows superior stability and performance at higher heat loads, with a maximum heat load capacity of 140 W.

These results indicate that P60F100 delivers superior thermal performance at low heat loads, whereas P60F150 provides more stable heat transfer performance under high heat load conditions. This demonstrates the critical importance of balancing the fill ratio and heat load in vapor chamber design and applications. Selecting the appropriate configuration based on specific requirements is essential to achieving optimal performance.

### 4.6. Comparison with Previous Additive Manufactured Vapor Chambers

Since no single paper is entirely identical to this study in terms of manufacturing process, materials, testing conditions, scope, and application objectives, a selection of partially related studies was chosen for comparison.

Several studies have explored additive manufacturing (AM) approaches for vapor chambers, each contributing to advancements in thermal performance.

Ozguc et al. [20] fabricated an additively manufactured vapor chamber (AMVC) using direct metal laser sintering (DMLS) with 316L stainless steel and compared its performance to a solid metal block. Their results showed significant thermal resistance reductions, similar to this study. However, their capillary structures were not optimized for heat transfer performance. In contrast, the TPMS Gyroid design in this study demonstrated improved liquid return and overall heat dissipation efficiency, enhancing the thermal management capabilities of vapor chambers. Meng et al. [21] developed an AMVC with a composite porous structure, incorporating TPMS Schwarz P-structured support columns and a TPMS Gyroid-structured condenser. Their findings indicated that AM-produced porous wicks enhanced capillary pumping capabilities, reducing temperature gradients. This study corroborates their results, particularly with P60F150 achieving better stability at high heat loads, highlighting the importance of fill ratio optimization in AMVC designs. Gu et al. [23] investigated grooved porous vapor chambers and AM-fabricated bionic wick structures. Their research showed significant improvements in liquid return efficiency, particularly when using bionic-inspired wick structures. This study aligns with their conclusions, emphasizing the role of capillary structure optimization in performance enhancement. However, the TPMS Gyroid design in this research further refines liquid return efficiency by ensuring uniformity and stability in the phase change process.

Zeng et al. [7] experimented with micro-grooved cavities (MGRA) for vapor chambers, demonstrating excellent anti-gravity capabilities and a thermal resistance of 0.055 K/W at a 30° tilt. While this study does not focus on orientation effects, it reinforces the importance of capillary-assisted phase change heat transfer, showing the benefits of structured porous wicks in various operational conditions.

Zhou et al. [26] enhanced SLM-fabricated Ω-shaped grooves, achieving a 10.5% reduction in thermal resistance and a 62.19% increase in thermal conductivity, optimizing heat transfer for microsatellites. Their study focused on lightweight, high-efficiency thermal management for space applications.

### 4.7. Comparison with Traditional Vapor Chamber Manufacturing

Traditional vapor chamber manufacturing techniques, such as sintering and machining, have also demonstrated significant improvements in thermal performance, albeit with limitations in precision and flexibility.

Deng et al. [5] achieved a 10–130% reduction in thermal resistance using radial grooves and sintered copper powder. However, their mechanically manufactured capillary structures were constrained by material limitations and bonding issues, leading to potential inefficiencies in liquid return. In contrast, the AM approach in this study eliminates these constraints, allowing for a seamless integration of complex capillary structures.

Liu et al. [6] demonstrated that multi-artery structures could achieve ultra-low thermal resistance (0.016 K/W). While this surpasses the results of this study, their method involved complex sintering and machining processes. This research, utilizing a monolithic AM approach, eliminates bonding defects and improves manufacturing efficiency while maintaining competitive thermal performance.

Zhou et al. [27] developed a leaf-vein-inspired vapor chamber with a cotton yarn wick, achieving 0.43 °C/W at 8.7 W. While their biomimetic design improved antigravity performance, manual fabrication limited scalability and precision.

Overall, this study’s findings highlight the potential of additive manufacturing in vapor chamber fabrication. By integrating TPMS Gyroid capillary structures, the study achieved a notable 75% reduction in thermal resistance compared to vapor chambers without capillary structures, showcasing the superiority of AM in enhancing heat dissipation efficiency. Future research should focus on further optimizing fill ratios and exploring alternative TPMS structures to maximize the performance benefits of AM-fabricated vapor chambers.

## 5. Conclusions

This study successfully employed additive manufacturing technology to fabricate an all-in-one vapor chamber and designed a capillary structure based on the TPMS Gyroid geometry to investigate its thermal performance under different fill ratios (P60F100 and P60F150). The results indicated that the sample with a higher fill ratio (P60F150) exhibited superior stability and thermal performance under high-power heat loads, while the lower fill ratio sample (P60F100) demonstrated excellent heat transfer performance under low-power conditions. By integrating precisely designed capillary structures with additive manufacturing, the study achieved a novel attempt at creating a vapor chamber through all-in-one fabrication.

- The advantages and limitations of horizontal and vertical printing in additive manufacturing for vapor chamber fabrication were identified. Vapor chambers typically require sufficient cavity volume for effective vapor circulation. Horizontal printing, while capable of reducing height, faces challenges due to large overhanging areas that increase the risk of printing failures. Adding more or larger support columns to address this issue compromises cavity volume. Vertical printing, on the other hand, is better suited for vapor chamber fabrication due to its smaller projected area when positioned vertically. This configuration increases the feasibility of mass production, as additive manufacturing has comparable preparation and post-processing requirements regardless of production scale. Printing more samples simultaneously significantly reduces the average production time per vapor chamber, enhancing its potential for practical applications.
- Under rigorous and realistic experimental conditions with a maximum temperature limit of 100 °C, it was confirmed that vapor chambers with capillary structures reduced thermal resistance by 75% compared to samples without capillary structures under identical fill ratios. The lowest thermal resistance of 0.3688 K/W was achieved at a heat load of 80 W, with the maximum heat load capacity reaching 140 W.
- The study compared vapor chambers with identical designs but different fill ratios (100% and 150%). The lower fill ratio sample exhibited a smaller overall thermal capacity, allowing for more pronounced boiling effects and lower stable temperatures and thermal resistance at low power levels, as long as the overall circulation remained manageable. However, with increasing power, the heating region showed signs of temperature rise around its periphery. This was attributed to the higher radial thermal resistance compared to axial thermal resistance, especially when low-thermal-conductivity metals were used. Once the heat transfer efficiency in the central region became insufficient to handle the heat load, the radial thermal resistance began to dominate, leading to an increase in overall thermal resistance. A higher fill ratio significantly delayed the onset of this phenomenon.

## Figures and Tables

**Table 1:** AMP-160 technical data.

**Max laser power**	300 W
**Building dimensions**	Ø160 mm × 160 mm
**Focus diameter**	50 μm
**Building volume**	1~10 cm^3^/h
**Scanning speed**	Max 1000 mm/s
**Layer thickness**	20~100 μm
**Size accuracy**	100 μm

**Table 2:** Laser parameters for the wall and wick.

	Wall	Wick
**Power (W)**	220	100
**Velocity (mm/s)**	900	1000
**Hatch (mm)**	0.1	0.1
**Thickness (mm)**	0.03	0.03
**Energy Density (J/mm^3^)**	81.5	33.3

**Table 3:** Detailed dimensions of vapor chambers.

Specifications of Vapor Chambers (Unit: mm)		
**Components of vapor chamber**		**Porosity (%)**
		60
**Dimensions of vapor chamber (L × W × H)**		62.5×62.5×5
**Dimensions of evaporator (L × W × H)**		62.5×62.5×1
**Wall thickness**		1
**Support and condenser thickness**		3
**Evaporator structures**		
**TPMS Gyroid**	**Unit cell size**	1×1×1
	**Thickness**	0.205

**Table 4:** Surface roughness parameters before and after grinding.

	$\mathrm{Before}\;\mathrm{Grinding}\;(\mu_{m}$)	$\mathrm{After}\;\mathrm{Grinding}\;(\mu_{m}$)
$S_{a,avg}$	18.5	2.0
$S_{q,avg}$	25.6	2.5
$S_{p,avg}$	61.3	18.1
$S_{v,avg}$	86.7	47.1
$S_{z,avg}$	147.9	65.3

**Table 5:** Sample filling amount and abbreviations.

Capillary Structure	Porosity (%)	Filling Rate (%)	Filling Amount (mL)	Abbreviation
Without wick	X	X	1.89	WVC
TPMS Gyroid	60	100	1.89	P60F100
		150	2.83	P60F150

**Table 6:** Experiment parameters for vapor chambers.

Experimental Parameters	
**Water temperature**	28 °C
**Water flow**	20 LPM
**Pressure**	20 kgf
**Sample**	WVC, P60F100, P60F150
**Heat load**	20 W, 40 W, 60 W, 80 W, 100 W, 120 W, 140 W
**Working fluid**	Methanol
**Extreme temperature**	100 °C

## Data Availability

The original contributions presented in the study are included in the article; further inquiries can be to the corresponding author.

## References

[1] Lin S., Lin D., Lin V., Shih T., Kang A., Wang Y.P. High Power Advanced Package with Water Cooling System Evaluation and Optimization. Proceedings of the 2023 22nd IEEE Intersociety Conference on Thermal and Thermomechanical Phenomena in Electronic Systems (ITherm).

[2] Lin P.Y., Kuo S.L., Yan K., Chen W.M., Liao D.D. Advanced Thermal Integration for HPC Packages with Two-Phase Immersion Cooling. Proceedings of the 2022 IEEE 72nd Electronic Components and Technology Conference (ECTC).

[3] Tsai M.C., Kang S.W., Paiva K. (2013). Experimental studies of thermal resistance in a vapor chamber heat spreader. Appl. Therm. Eng..

[4] Tang Y., Deng D., Lu L., Pan M., Wang Q. (2010). Experimental investigation on capillary force of composite wick structure by IR thermal imaging camera. Exp. Therm. Fluid Sci..

[5] Deng D., Huang Q., Xie Y., Huang X., Chu X. (2017). Thermal performance of composite porous vapor chambers with uniform radial grooves. Appl. Therm. Eng..

[6] Liu C., Hu D., Li Q., Chen X., Zhang Z., Zhou F. (2021). Vapor chamber with two-layer liquid supply evaporator wick for high-heat-flux devices. Appl. Therm. Eng..

[7] Zeng J., Zhang S., Chen G., Lin L., Sun Y., Chuai L., Yuan W. (2018). Experimental investigation on thermal performance of aluminum vapor chamber using micro-grooved wick with reentrant cavity array. Appl. Therm. Eng..

[8] Li B., Yin X., Tang W., Zhang J. (2020). Optimization design of grooved evaporator wick structures in vapor chamber heat spreaders. Appl. Therm. Eng..

[9] Tang Y., Yuan D., Lu L., Wang Z. (2013). A multi-artery vapor chamber and its performance. Appl. Therm. Eng..

[10] Yan C., Li H., Tang Y., Ding X., Yuan X., Liang Y., Zhang S. (2023). A novel ultra-thin vapor chamber with composite wick for portable electronics cooling. Appl. Therm. Eng..

[11] Hassanin H., Elshaer A., Benhadj-Djilali R., Modica F., Fassi I. (2018). Surface Finish Improvement of Additive Manufactured Metal Parts, Micro and Precision Manufacturing. Micro and Precision Manufacturing. Engineering Materials.

[12] Jadhav S.D., Goossens L.R., Kinds Y., Hooreweder B.V., Vanmeensel K. (2021). Laser-based powder bed fusion additive manufacturing of pure copper. Addit. Manuf..

[13] Sajjad U., Rehman T., Ali M., Park C.W., Tan W.M. (2022). Manufacturing and potential applications of lattice structures in thermal systems: A comprehensive review of recent advances. Int. J. Heat Mass Transf..

[14] Frazier W.E. (2014). Metal Additive Manufacturing: A Review. J. Mater. Eng. Perform..

[15] Zhang J., Lian L., Liu Y. (2020). Liquid phase enhanced sintering of porous aluminum for cylindrical Al-acetone heat pipe. Int. J. Heat Mass Transf..

[16] Kaur I., Singh P. (2020). Flow and Thermal Transport Through Unit Cell Topologies of Cubic and Octahedron Families. Int. J. Heat Mass Transf..

[17] Gan Z., Turner M.D., Gu M. (2016). Biomimetic Gyroid Nanostructures Exceeding Their Natural Origins. Sci. Adv..

[18] Han L., Che S. (2018). An Overview of Materials with Triply Periodic Minimal Surfaces and Related Geometry: From Biological Structures to Self-Assembled Systems. Adv. Mater..

[19] Al-Ketan O., Rowshan R., Al-Rub R.K.A. (2018). Topology-mechanical property relationship of 3D printed strut, skeletal, and sheet based periodic metallic cellular materials. Addit. Manuf..

[20] Ozguc S., Pai S., Pan L., Geoghegan P.J., Weibel J.A. Experimental Demonstration of an Additively Manufactured Vapor Chamber Heat Spreader. Proceedings of the 2019 18th IEEE Intersociety Conference on Thermal and Thermomechanical Phenomena in Electronic Systems (ITherm).

[21] Meng X., Tan S., Yuan Z., Zhang Y., Chen L. (2023). Experimental Study on the Heat Transfer Performance of a Vapour Chamber with Porous Wick Structures Printed Via Metallic Additive Manufacturing. Int. Commun. Heat Mass Transf..

[22] Gu Z., Liu H., Yang K., Wang Q., Xu H., Zhang L. (2023). Enhancing Heat Transfer Performance in 3D-Printed Integrated Vapor Chamber Using Composite Structures. Appl. Therm. Eng..

[23] Gu Z., Yang K., Liu H., Zhou X., Xu H., Zhang L. (2024). Enhancing Heat Transfer Performance of Aluminum-Based Vapor Chamber with a Novel Bionic Wick Structure Fabricated Using Additive Manufacturing. Appl. Therm. Eng..

[24] Yap C.Y., Chua C.K., Dong Z.L., Liu Z.H., Zhang D.Q., Loh L.E., Sing S.L. (2015). Review of selective laser melting: Materials and applications. Appl. Phys..

[25] Chen K.L., Luo K.Y., Kang S.W. (2023). SLM Additive Manufacturing of Oscillating Heat Pipe. Sustainability.

[26] Zhou J., Teng L., Shen Y., Jin Z. (2023). Simulation of, Optimization of, and Experimentation with Small Heat Pipes Produced Using Selective Laser Melting Technology. Materials.

[27] Zhou Z., Wang X., Zhou Y. (2023). Performance Study of a Leaf-Vein-like Structured Vapor Chamber. Materials.

